# Neuromarkers and neurological outcome in out-of-hospital cardiac arrest patients treated with therapeutic hypothermia–experience from the HAnnover COoling REgistry (HACORE)

**DOI:** 10.1371/journal.pone.0245210

**Published:** 2021-01-07

**Authors:** Muharrem Akin, Vera Garcheva, Jan-Thorben Sieweke, John Adel, Ulrike Flierl, Johann Bauersachs, Andreas Schäfer

**Affiliations:** Cardiac Arrest Centre, Department of Cardiology and Angiology, Hannover Medical School, Hannover, Germany; Maastricht University Medical Center, NETHERLANDS

## Abstract

**Background:**

Neuron-specific enolase (NSE) and S-100b have been used to assess neurological damage following out-of-hospital cardiac arrest (OHCA). Cut-offs were derived from small normothermic cohorts. Whether similar cut-offs apply to patients treated with hypothermia remained undetermined.

**Methods:**

We investigated 251 patients with OHCA treated with hypothermia but without routine prognostication. Neuromarkers were determined at day 3, neurological outcome was assessed after hospital discharge by cerebral performance category (CPC).

**Results:**

Good neurological outcome (CPC≤2) was achieved in 41%. Elevated neuromarkers, older age and absence of ST-segment elevation after ROSC were associated with increased mortality. Poor neurological outcome in survivors was additionally associated with history of cerebrovascular events, sepsis and higher admission lactate. Mean NSE was 33μg/l [16–94] vs. 119μg/l [25–406]; p<0.001, for survivors vs. non-survivors, and 21μg/l [16–29] vs. 40μg/l [23–98], p<0.001 for good vs. poor neurological outcome. S-100b was 0.127μg/l [0.063–0.360] vs. 0.772μg/l [0.121–2.710], p<0.001 and 0.086μg/l [0.061–0.122] vs. 0.138μg/l [0.090–0.271], p = 0.009, respectively. For mortality, thresholds of 36μg/l for NSE and 0.128μg/l for S-100b could be determined; for poor neurological outcome 33μg/l (NSE) and 0.123μg/l (S-100b), respectively. Positive predictive value for NSE was 81% (74–88) and 79% (71–85) for S-100b.

**Conclusions:**

Thresholds for NSE and S-100b predicting mortality and poor neurological outcome are similar in OHCA patients receiving therapeutic hypothermia as in those reported before the era of hypothermia. However, both biomarkers do not have enough specificity to predict mortality or poor neurological outcome on their own and should only be additively used in clinical decision making.

## Introduction

Following cardio-pulmonary resuscitation (CPR) and return of spontaneous circulation (ROSC) after out-of-hospital cardiac arrest (OHCA), the first 24 hours critically determine the extent of brain damage [1]. Therapeutic hypothermia with a target temperature of 32–34°C had been established for treatment of patients with OHCA to reduce oxygen consumption and demand by the brain. In the randomised-controlled Hypothermia After Cardiac Arrest trial lowering body temperature to 33°C for 24 hours reduced mortality and improved neurological outcome at 6 months [2]. Another study simultaneously demonstrated a mortality reduction by therapeutic hypothermia and increased direct hospital discharge [3].

Therefore, therapeutic hypothermia is considered superior to no temperature control regarding mortality after OHCA and recommended in comatose OHCA survivors [4]. As “non-hypothermic” patients had been frequently subfebrile [3] and patients with post-hypothermia fever show higher mortality and less favourable neurological outcome [5], the Targeted Temperature Management (TTM)-trial compared controlled “targeted” temperature of 36°C to hypothermia of 33°C [6]. Overall mortality in the TTM-trial was higher than in trials performed ten years earlier. One major concern regarding the TTM-trial was the use of routine neurological prognostication, which is not well evaluated for patients during or early after therapeutic hypothermia. Several analyses have been reported from the TTM-trial regarding neuromarkers, however, due to frequent withdrawal of life support based on routine prognostication, their predictive value regarding neurological outcome is unclear [7, 8]. Of neuromarkers, neuron-specific enolase (NSE) and S-100b serum levels are widely used to provide at least some impression on neurological damage. However, their predictive values regarding poor neurological outcome has mostly been derived from patient cohorts before therapeutic hypothermia was implemented and was based on smaller populations [9, 10]. Therefore, the question whether similar decision thresholds apply for these neuromarkers in patients treated with therapeutic hypothermia remains unresolved. Use of routine prognostication intending to withdraw life-support early clearly impacts outcome. Hence, a careful analysis of neurological outcome in OHCA survivors treated with therapeutic hypothermia without routine withdrawal of life-support based on routine prognostication is still warranted.

We previously implemented an advanced and defined algorithm of standardised interdisciplinary assessment of OHCA patients in our hospital, the Hannover Cardiac Resuscitation Algorithm (HaCRA) [11], which includes all requirements for treating OHCA patients as recently stated by the German Resuscitation Council [12] in line with current guidelines on post-resuscitation care [13]. Our strategy aims for early diagnosis and treatment of potentially life threatening conditions and–in contrast to the TTM-trial- explicitly excludes a standardised prognostication procedure. Therefore, our patient population provides an excellent cohort to study whether there is a different cut-off level for prognostic assessments using those neuromarkers. Here we report the outcome of patients treated by HaCRA in relation to their observed NSE and S-100b serum levels.

## Methods

### Study design and patients

The HAnnover COoling REgistry -HACORE- is a prospective observational registry including all OHCA patients admitted to the cardiac arrest centre at Hannover Medical School. All patients were treated with therapeutic hypothermia according to HaCRA [11], which does not include routine prognostication. Neuromarkers of patients admitted between 01/2011 and 12/2016 were determined at day 3 after admission. Out of 302 patients admitted, 51 (17%) were excluded from the neuromarker analysis because they had already died before day 3. 251 patients (83%) were available for further analysis. The primary outcome assessment is 30-day mortality; secondary outcome is poor neurological function in survivors or death defined as Cerebral Performance Category (CPC) ≥3 on the day of discharge from either inpatient medical treatment or intensive care neurological rehabilitation, which followed inpatient treatment in patients with persistent neurological deficit [14].

### OHCA management using HaCRA

Patients after OHCA were first screened and stabilised in the emergency room after primarily successful CPR. The standard includes immediate endotracheal airway management in case of a primary laryngeal tube or mask, continuation of mechanical CPR by an automated compression device in case of ongoing CPR, and early determination of cardiac function by transthoracic echocardiography in patients with ROSC. In case of cardiogenic shock, complete revascularisation was attempted as recommended by guidelines at that time [15] along with active hemodynamic support if needed [16, 17]. To perform therapeutic hypothermia on intensive care unit (ICU), an intravascular cooling catheter (Coolguard Quattro®, ZOLL Medical, San Jose, CA, USA) was placed in the right femoral vein. An active cooling device was chosen to select and maintain a constant target temperature of 32°C for 24 hours followed by controlled rewarming (0.25°C per hour) and normothermia for another 72 hours [11]. After haemodynamic stabilisation in acute cardiac intensive care, early transfer for neurological-intensive care rehabilitation was intended if patients remained comatose after rewarming.

Initial pH and lactate were determined from first arterial blood gas analysis collected in the emergency room prior to any treatment. Neuromarkers were determined from clinical routine blood samples taken at day 3 after admission using standard serum tubes by immune assay (Roche, ECLIA kit test (Electro-Chemi-Luminescent-Immuno Assay); Roche Diagnostics GmbH, Switzerland for NSE and Cobas 8000 auto-analyzer; Roche Diagnostics GmbH, Mannheim, Germany for S-100b). Even so clinicians had knowledge of neuromarker values, at no time was the decision to discontinue life-supporting therapy made solely on the basis of these values. In contrast, elevated neuromarkers were only considered to trigger further diagnostics or re-evaluations, e.g. by obtaining new cranial imaging.

### Clinical follow-up

Patients were followed up for the period of hospital stay and data were extracted from electronic hospital patient data management systems. Discharge letters from rehabilitation facilities were collected.

### Ethics

The HAnnover COoling REgistry -HACORE- is a prospective observational registry including anonymized data from all OHCA patients treated at our cardiac arrest centre. HACORE is approved by the ethics committee at Hannover Medical School (#3567–2017) and is in accordance with the Declaration of Helsinki. The ethics committee approved the analysis as reported in the present manuscript. Written informed consent was obtained from legal guardians during the unconscious period and re-consented by survivors after gaining consciousness.

### Statistical analysis

Baseline characteristics are presented as frequencies (n) and percentages (%), means ± standard deviation (SD) for normally distributed variables, or median and interquartile ranges (IQR) for non-normally distributed variables. Normally distributed variables were compared by Student´s t-test and Mann-Whitney test for nonparametric data, respectively. All group comparisons of continuous measures were performed using Wilcoxon’s test, whereas the chi-square or Fisher’s exact test was used to assess categorical data. Cumulative mortality was estimated by Kaplan-Meier method with statistical significance examined by the log-rank test. Univariate Cox regression was performed including all variables potentially associated with 30-day mortality. Predictors of mortality were specified using a multivariate Cox regression analysis with those variables, which were statistically significant in univariate Cox regression analysis. A similar analysis was performed for neurological outcome after hospital discharge by logistic regression analysis. Results from the regression analyses were displayed as hazard ratios (HRs) with 95% confidence intervals (CIs). Prior multicollinearity was assessed by variance inflation factor (VIF). We assessed the predictive accuracy of NSE and S-100b for mortality and good neurological outcome according to CPC-score by receiver operating characteristic (ROC) curves. Results were expressed in terms of area under the curve (AUC) and 95% CI for this area. Cut-off values for prediction were defined as the cut-off point having the highest Youden index (Yi = sensitivity + specificity -1). Sensitivity, specificity, positive and negative predictive value, accuracy for determined cut-offs were calculated as well as different levels of false positive rates and their sensitivity to predict poor outcome with thresholds for NSE and S-100b for comparison with previous studies.

Statistical analyses were performed using SPSS Statistics 24 (IBM SPSS Statistics 24) and GraphPad Prism 6.0 (GraphPad Software, Inc., La Jolla, CA) for creating figures. A two-sided p-value of <0.05 was considered statistically significant.

## Results

### Study population

In this real-world cohort of 251 patients, 83 (33%) died within the first 30 days (Table 1). Good neurological outcome categorized as CPC≤2 was achieved in 102 (41%) patients.

**Table 1:** Demographic data for all patients surviving first 3 days after admission investigated in the HAnnover COoling REgistry (HACORE). Characteristics are also shown divided in survivors and non survivors within day 3–30 and good (CPC 1 and 2) to poor (CPC 3,4 and 5) outcome.

Demographics	HACORE all patients	HACORE non-survivors	HACORE Survivors	p value	HACORE–good outcome (CPC ≤ 2)	HACORE–poor outcome (CPC ≥ 3)	p value
Number (%)	251 (100)	83 (33)	168 (67)		102 (41)	149 (59)	
Age–years (mean±SD)	62±16	68±12	59±13	**<0.001**	57±13	66±13	**<0.001**
Male sex (%)	197 (78)	34 (43)	134 (80)	0.293	81 (79)	116 (78)	0,876
**Cardiovascular risk factors**							
Arterial hypertension (%)	141 (56)	47 (57)	94 (56)	1.000	46 (45)	95 (64)	**0,004**
Diabetes mellitus (%)	58 (23)	26 (31)	32 (19)	**0.038**	17 (17)	41 (28)	**0.048**
Hyperlipidaemia (%)	87 (35)	25 (30)	62 (37)	0.325	35 (34)	52 (35)	1,000
Family history for CAD (%)	28 (11)	4 (5)	24 (14)	**0.032**	16 (16)	12 (8)	0.068
Nicotine abusus (%)	98 (39)	24 (29)	74 (44)	**0.027**	50 (49)	48 (32)	**0.007**
**Previous comorbidities**							
CAD (%)	57 (23)	19 (23)	38 (23)	1.000	22 (22)	35 (24)	0.761
PCI (%)	32 (13)	9 (11)	23 (14)	0.688	12 (12)	20 (13)	0.848
CABG (%)	21 (8)	8 (10)	13 (8)	0.632	7 (7)	14 (9)	0.643
PAD (%)	20 (8)	12 (14)	8 (5)	**0.012**	6 (6)	14 (9)	0.352
TIA/stroke (%)	27 (11)	16 (19)	11 (7)	**0.004**	5 (5)	22 (15)	**0.013**
Atrial fibrillation (%)	52 (21)	23 (28)	29 (17)	0.068	16 (16)	36 (24)	0.115
Asthma or COPD (%)	24 (10)	16 (19)	8 (5)	**<0.001**	2 (2)	22 (15)	**<0.001**
Chronic kidney disease (%)	34 (14)	21 (25)	13 (8)	**<0.001**	8 (8)	26 (17)	**0.038**
Renal replacement therapy (%)	3 (1)	2 (2)	1 (1)	0.255	0 (0)	3 (2)	0.273
**Characteristics of cardiac arrest**							
ROSC, min (mean±SD)	22±16	24±16	22±16	0.388	21±16	23±16	0.531
Shockable primary rhythm (%)	186 (74)	54 (65)	132 (79)	**0.031**	81 (79)	105 (71)	0.142
- Defibrillations (IQR)	3 [1–4]	3 [0–4]	3 [1–4]	0.586	2 [1–4]	2 [1–4]	0.120
ST seg. elevation after ROSC (%)	118 (47)	30 (36)	88 (52)	**0.016**	56 (55)	62 (42)	**0.041**
Witnessed arrest (%)	214 (85)	65 (78)	149 (89)	**0.037**	92 (90)	122 (82)	0.073
Bystander CPR (%)	172 (69)	52 (63)	120 (71)	0.193	77 (72)	95 (64)	0.054
Ongoing CPR	20 (8)	9 (10)	11 (7)	0.321	8 (8)	12 (8)	1.000
**Characteristics of hospital stay**							
Temperature at admission (°C) (IQR)	35 [34–36]	34.7 [33.7–36.6]	34.6 [33.6–36.9]	0.642	35 [33–36]	35 [34–36]	0.076
SAPS II score (mean±SD)	51±11	62±11	49±11	**<0.001**	48±10	53±11	**0.006**
Mechanical ventilation (days) (IQR)	10 [6–18]	11 [6–17]	11 [7–19]	0.081	9 [6–17]	11 [7–18]	0.320
Duration of hospital stay (days) (IQR)	14 [9–20]	10 [5–16]	17 [11–21]	**<0.001**	16 [12–22]	11 [7–17]	**<0.001**
Renal replacement therapy (%)	65 (26)	32 (39)	33 (20)	**0.002**	16 (16)	49 (33)	**0.002**
Sepsis (%)	66 (26)	37 (45)	29 (17)	**<0.001**	16 (16)	50 (34)	**0.002**
Cardiogenic shock (%)	138 (55)	47 (57)	91 (54)	0.685	51 (50)	87 (58)	0.196
Refractory CS (%)	42 (17)	22 (27)	20 (12)	**0.004**	12 (12)	30 (20)	0.085
**Mechanical assist device**							
Impella microaxial pump (%)	41 (16)	18 (22)	23 (14)	0.146	14 (14)	27 (18)	0.389
va-ECMO (%)	18 (7)	8 (10)	10 (6)	0.305	7 (7)	11 (7)	1.000
IABP (%)	4 (2)	0 (0)	4 (2)	0.305	2 (2)	2 (1)	1.000
**Catheterisation laboratory**							
Coronary angiography (%)	243 (97)	78 (94)	165 (98)	**0.102**	100 (98)	143 (96)	0.479
PCI (%)	148 (59)	45 (54)	103 (61)	0.340	65 (64)	83 (56)	0.240
**Laboratory values**							
Admission lactate (mmol/l) (IQR)	6.5 [3.4–9.2]	7.9 [4.9–14.7]	6.3 [2.9–8.4]	**0.011**	4.8 [2.6–8.3]	6.9 [4.3–9.8]	**0.003**
Admission pH (IQR)	7.24 [7.10–7.31]	7.18 [7.08–7.44]	7.21 [7.12–7.32]	0.089	7.28 [7.17–7.34]	7.20 [7.07–7.29]	**0.004**
NSE on day 3 (μg/l)* (IQR)	27 [18–58]	119 [25–406]	33 [16–94]	**<0.001**	21 [16–29]	40 [23–98]	**<0.001**
S-100b on day 3 (μg/l)* (IQR)	0.117 [0.074–0.200]	0.772 [0.121–2.710]	0.127 [0.063–0.360]	**<0.001**	0.086 [0.061–0.122]	0.138 [0.090–0.271]	**0.009**

Data are presented as absolute numbers and percentages, mean±SD or median and lower and upper quartile (IQR) as appropriate. The p value represents comparison between groups of non-survivors and survivors, and of survivors with good and poor neurological outcome after medical discharge. Abbreviations: CABG–coronary artery bypass graft; CAD–coronary artery disease; COPD–chronic obstructive pulmonal disease; CPR–cardiopulmonary resuscitation; CS–cardiogenic shock; HACORE–HAnnover COoling REgistry; ROSC–return of spontaneous circulation; IABP–intra-aortic balloon pump; IQR–Interquartile range; NSE—neuronspecific enolase; PAD–periphery artery disease PCI–percutaneous coronary intervention; S-100b –protein S-100b; TIA–transient ischemic attack; va-ECMO–veno-arterial extracorporeal membrane oxygenation

*measured at day 3 after resuscitation

### Baseline characteristics, predictors for mortality and neurological outcome

Patient characteristics are summarized in Table 1. In multivariate analysis older age, absence of ST-segment elevation after ROSC as well as elevated neuromarkers were associated with increased mortality (Table 2). Poor neurological outcome was associated with older age, history of cerebrovascular events, absence of ST-segment elevation after ROSC, sepsis, increased admission lactate as well as elevated neuromarkers (Table 3). In relation to 30-day survival, the levels of both biomarkers were significantly higher on day 3 in patients who did not survive. On the same day of assessment, in survivors the levels of both biomarkers were significantly higher in patients with poor neurological outcomes compared to those with good neurological outcome (Table 1). Regarding the value of neuromarkers there was a difference in patients related to the cause of death with significantly higher values in patients with anoxic brain damage compared to those with other aetiologies (Table 4). Since almost half of deaths were un-related to anoxic brain damage, this at least partly explains some CPC 5 events in the lower neuromarker quartiles.

**Table 2:** Univariate and multivariate cox regressions analysis for mortality at 30-day follow-up in the HAnnover COoling Registry (HACORE).

Demographics	Univariate analysis			Multivariate analysis		
	HR	(95% CI)	p value	HR	(95% CI)	p value
Age	1.056	(1.036–1.076)	0.001	1.042	(1.017–1.067)	0.001
Arterial hypertension	1.002	(0.649–1.546)	0.994			
Diabetes mellitus	1.631	(1.026–2.595)	0.039			
Nicotine abusus	0.567	(0.352–0.911)	0.019			
TIA/stroke	2.347	(1.359–4.054)	0.002			
Asthma or COPD	2.978	(1.724–5.146)	0.001			
Chronic kidney disease	2.615	(1.592–4.295)	0.001			
ST seg. elevation after ROSC	0.577	(0.369–0.904)	0.016	0.510	(0.297–0.874)	0.014
SAPS II score	1.034	(1.014–1.054)	0.001			
Admission lactate	1.064	(1.018–1.112)	0.006			
Admission pH	0.313	(0.088–1.109)	0.072			
NSE*	1.007	(1.005–1.008)	0.001	1.005	(1.003–1.007)	0.001
S-100b*	1.365	(1.234–1.510)	0.001	1.208	(1.050–1.389)	0.008

NSE and S-100b are associated with 30-day mortality as well as age and ST segment elevation after ROSC in multivariate Cox regression analysis.

*measured at day 3 after resuscitation

**Table 3:** Univariate and multivariate regressions analysis for poor (CPC ≥3) outcome in the HAnnover COoling Registry (HACORE).

Demographics	Univariate analysis			Multivariate analysis		
	HR	(95% CI)	p value	HR	(95% CI)	p value
Age	1.040	(1.026–1.054)	0.001	1.032	(1.014–1.050)	0.001
Arterial hypertension	1.367	(0.979–1.909)	0.067			
Diabetes mellitus	1.426	(0.995–2.043)	0.053			
Nicotine abusus	0.634	(0.450–0.895)	0.009			
TIA/stroke	1.993	(1.265–3.139)	0.003	1.961	(1.108–3.470)	0.021
Asthma or COPD	2.505	(1.591–3.945)	0.001			
Chronic kidney disease	22.674	(0.851–8.400)	0.092			
ST seg. elevation after ROSC	0.698	(0.504–0.967)	0.031	0.651	(0.44–0.952)	0.027
Duration of hospital stay	0.923	(0.898–0.948)	0.001			
Renal replacement therapy	1.772	(1.258–2.495)	0.001			
Sepsis	1.984	(1.410–2.790)	0.001	1.552	(1.020–2.361)	0.040
Admission lactate	1.051	(1.017–1.087)	0.003	1.066	(1.001–1.136)	0.048
Admission pH	0.298	(0.117–0.759)	0.011			
NSE*	1.007	(1.005–1.008)	0.001	1.004	(1.003–1.006)	0.001
S-100b*)	1.369	(1.238–1.513)	0.001	1.217	(1.057–1.402)	0.006

NSE and S-100b are associated with poor neurological outcome (CPC≥3) as well as age, history of cerebrovascular events, ST segment elevation after ROSC and sepsis, lactate at admission in multivariate regression analysis.

*measured at day 3 after resuscitation

**Table 4:** Distribution of neuromarkers according to the cause of death.

	n = 83 (%)	NSE on day 3 (μg/l)	p value	S-100b on day 3 (μg/l)	p value
**Anoxic brain damage**	40 (48)	104 [78–262]	**<0.001**	0.460 [0.211–1.725]	**0.004**
**Non-anoxic brain damage**	43 (52)	32 [23–49]		0.138 [0.100–0.203]	
			0.633		0.429
Septic shock (%) (IQR)	18 (22)	32 [24–67]		0.152 [0.113–0.208]	
Cardiogenic shock (%) (IQR)	9 (11)	25 [17–47]		0.128 [0.100–0.147]	
MODS (%) (IQR)	15 (18)	36 [22–48]		0.164 [0.080–0.241]	
ICB (%) (IQR)	1 (1)	36		0.075	

Data are represented in absolute numbers and percentages, median, lower and upper quartile (IQR) for NSE and S-100b determined on day 3. MODS–multi organ dysfunction syndrome; ICB–intra cranial bleeding

### ROC analysis for cut-offs and thresholds for NSE and S-100b regarding mortality and poor neurological outcome

**Fig 1:**
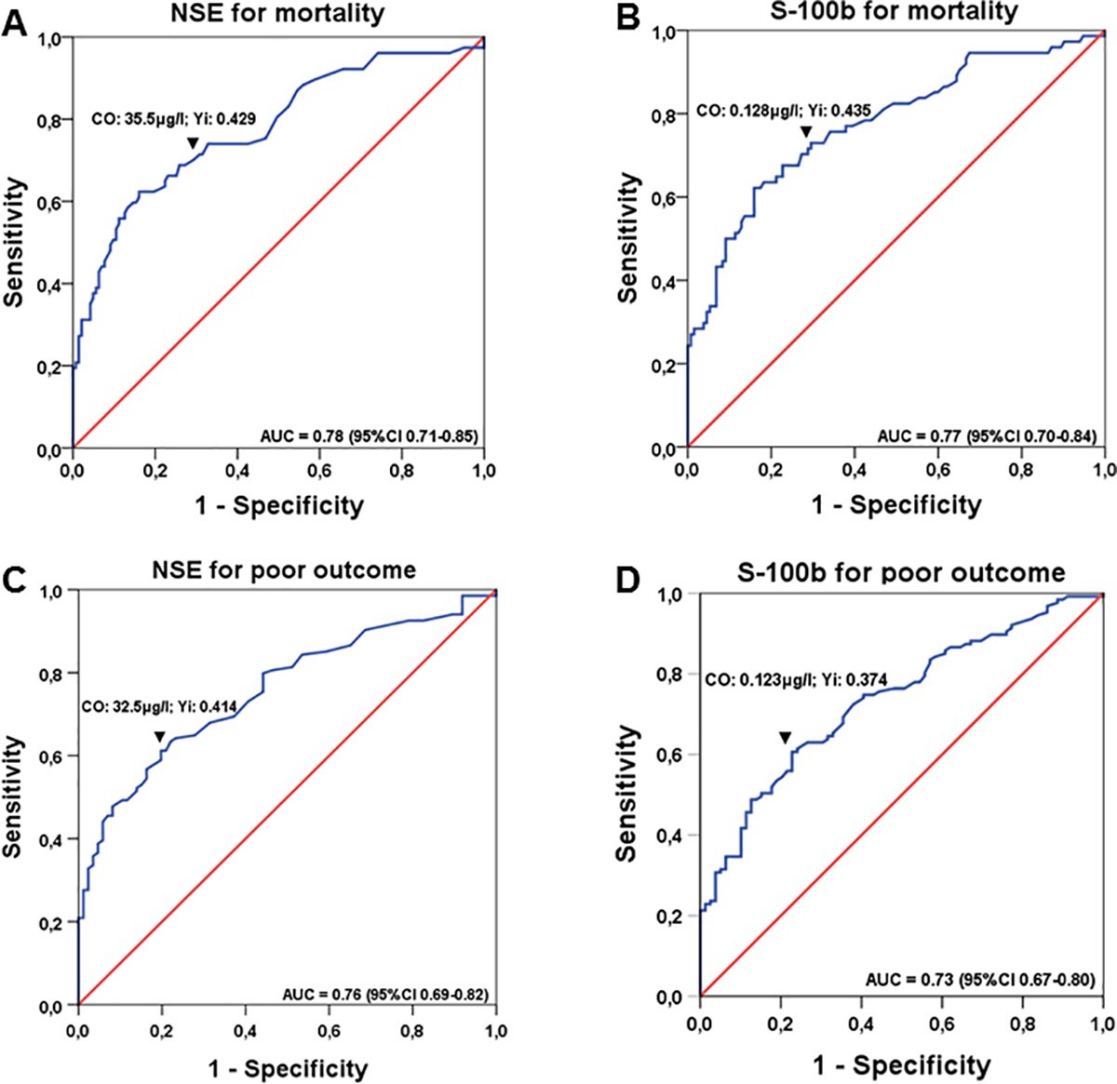
ROC curves for determining cut-off values of NSE and S-100b for mortality and neurological outcome: ROC analysis for NSE (A and C) and S-100b (B and D) are presented for mortality (A and B), and neurological outcome (C and D), respectively, with corresponding areas under the curve (AUC) and confidence-intervals (CI). Cut-off values (co) identified by highest Youden-index (Yi) are shown above downwards pointing arrowheads (▼).

ROC analyses for NSE (Fig 1A & 1C) and S-100b (Fig 1B & 1D) were performed for mortality (Fig 1A & 1B) and poor neurological outcome (Fig 1C & 1D). The AUC to predict mortality was 0.78 (95%CI 0.71–0.85) for NSE with a cut-off value of 35.5 μg/l (sensitivity 68.8%; specificity 74.1%), and 0.77 (95%CI 0.70–0.84) for a cut-off value of 0.128 μg/l (sensitivity 73.0%; specificity 70.5%) for S-100b, respectively.

To predict poor neurological outcome (including mortality), AUC was 0.76 (95%CI 0.69–0.82) with a cut-off value of 32.5 μg/l for NSE (sensitivity 61.2%; specificity 80.2%) and 0.73 (95%CI 0.67–0.80) for a cut-off value of 0.123 μg/l for S-100b (sensitivity 61.4%; specificity 76.0%) (Table 5).

**Table 5:** Sensitivity, specificity, positive predictive value, negative predictive value and accuracy for predicting poor neurological outcome (CPC≥3) according to determined cut off values for NSE and S-100b.

	Poor neurological outcome (CPC≥3)	
	NSE	S-100b
	32.5 μg/l	0.123 μg/l
Sensitivity*	61.2 (48.0–65.9)	61.4 (50.6–68.8)
Specificity*	80.2 (70.3–88.0)	76.0 (65.0–84.9)
PPV*	80.9 (72.9–86.9)	79.1 (71.4–85.2)
NPV*	56.1 (50.5–61.6)	55.6 (49.3–61.7)
Accuracy*	66.5 (59.7–72.8)	66.3 (59.3–72.9)

Sensitivity, Specificity, Positive Predictive Value (PPV), Negative Predictive Value (NPV) and Accuracy given in *% with according confidence intervals of determined cut off values for NSE and S-100b predicting poor neurological outcome (CPC≥3)

Although predictive values of NSE and S-100b for mortality and poor neurological outcome itself are similar, in combination they are more reliable (for mortality: sensitivity 75.5%; specificity 80.0% and for poor neurological outcome: sensitivity 66.7%; specificity 90.0%, respectively).

Thresholds for NSE to predict poor outcome at a FPR level of 0% was 106μg/l (sensitivity: 32.2% (95%CI 24.8–40.4)) and at a level of 5% 47μg/l (sensitivity: 53.7% (95%CI 45.4–61.9)), For S-100b, 0% FPR was at 0.374μg/l (sensitivity: 34.9% (95%CI 27.3–43.1)) and 5% FPR at 0.218μg/l (sensitivity 45.6% (95%CI 37.5–54.0)) (Table 6a and 6b).

**Table 6:** False positive rate, sensitivity, specificity and corresponding NSE (a) and S-100b (b) for prediction of poor neurological outcome (CPC≥3).

**a)**			
**FPR in %**	**Sensitivity in %**	**Specificity in %**	**NSE, μg/l**
0	32.2 (24.8–40.4)	100 (96.5–100)	106
1	38.3 (30.5–46.6)	99.1 (94.7–99.9)	92
2	43.0 (34.9–51.3)	98.0 (93.1–99.8)	78
3	45.6 (37.5–54.0)	97.1 (91.6–99.4)	73
4	48.3 (40.1–56.6)	96.1 (90.3–98.9)	58
5	53.7 (45.4–61.9)	95.1 (88.9–98.4)	47
**b)**			
**FPR in %**	**Sensitivity in %**	**Specificity in %**	**S-100b, μg/l**
0	34.9 (27.3–43.1)	100 (96.5–100)	0,374
1	35.6 (27.9–43.8)	99.1 (94.6–99.9)	0,360
2	36.2 (28.5–44.5)	98.0 (93.1–99.7)	0,320
3	42.3 (34.2–50.6)	97.0 (91.8–99.4)	0,243
4	42.9 (34.9–51.3)	96.1 (90.3–98.2)	0,239
5	45.6 (37.5–54.0)	95.1 (88.9–98.4)	0,218

False positive Rate (FPR), Sensitivity, Specificity with confidence intervals according to thresholds for NSE and S-100b for prediction of poor neurological outcome (CPC≥3)

### CPC distribution in relation to neuromarker levels and cut-off values

**Fig 2:**
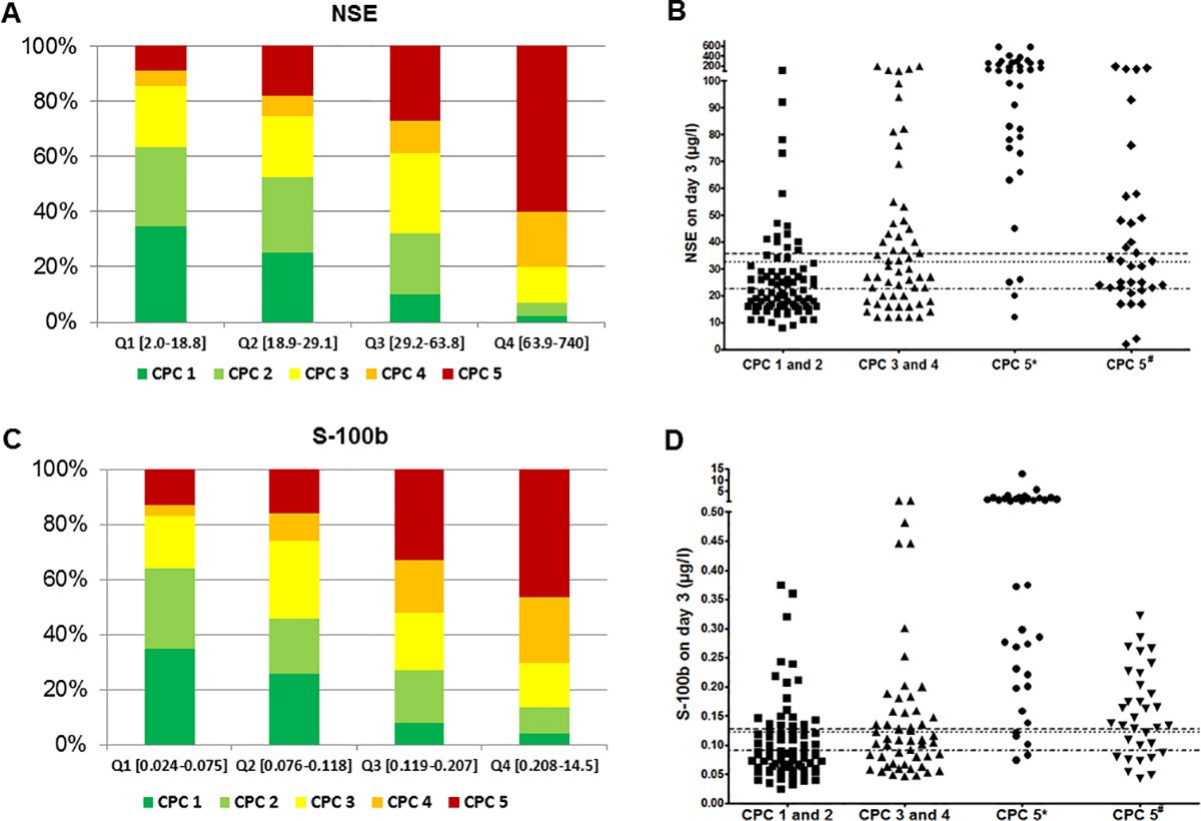
Distribution of CPC-classes according to interquartiles of NSE and S-100b and relation to cut-off values: Proportions of CPC classes according to the interquartile distributions of NSE (A) and S-100b (C), respectively, and scatter plots of survivors with good (CPC 1 and 2) and poor neurological outcome (divided in CPC 3 and 4 and CPC 5 separated by *anoxic and ^#^other causes of death) for NSE (B) and S-100b (D) with cut-off values for mortality (dashed line) and poor neurological outcome (dotted line) and poor neurological outcome of survivors (dotted and dashed line).

Lower NSE and S-100b values were associated with more favourable neurological outcome (Fig 2A for NSE and Fig 2C for S-100b). The illustration of individual levels of NSE (Fig 2B) and S-100b (Fig 2D) in relation to CPC categories shows that both markers are not suitable to predict outcome on an individual basis. If the ROC-determined cut-off for poor neurological outcome were used, for NSE 19.8% (95%CI 12.0–28.7) and for S-100b 24.0% (95%CI 15.1–35.0) of patients actually achieving a good neurological outcome would be falsely predicted a poor outcome (Table 5). Even when combining both neuromarkers, the false positive ratio (FPR) was 10.4% (95%CI 4.7–21.1). When using cut-offs for mortality, for NSE 27.4% (95%CI 20.1–35.0) and for S-100b 31.3% (95%CI 23.2–39.4) of patients actually achieving a good outcome would be predicted incorrectly. Also, when combining both, FPR was more favourable in combination (20% (95%CI 13–30)), but still unacceptably high.

## Discussion

When applying a hypothermia protocol without standardized withdrawal of life-support, we found that the overall relation between biomarker levels and neurological outcome seems to be similar to historic values reported from cohorts prior to using hypothermia. Nevertheless, on an individual basis, the predictive values of NSE and S-3100b are not high enough to use them solitarily. Extremely high levels of those markers might help to guide further imaging or neurological examinations [18].

Mortality and good neurological outcome after OHCA in a real life population is dependent on rapid recognition of arrest, bystander-initiated basic-life support and high-quality out-of-hospital and in-hospital care. Even when using standardised intrahospital protocols, outcome is relevantly determined by extra-hospital care provided before. Biomarkers like NSE and S-100b are used to predict outcome such as mortality and neurological performance. After OHCA mortality is still high and the neurological outcome is often poor [19, 20]. While cardiovascular instability is the main cause of mortality in the first 3 days after OHCA, impaired neurological function becomes more important thereafter [19, 21].

As long term prognosis of OHCA survivors is essentially determined by their neurological status, an early assessment is crucial to predict an unfavorable course with poor neurological outcome [22]. Anoxic brain injury is the leading neurological cause of death following OHCA rather than brain death, which accounts for only about 10% of causes [19, 21]. Unfortunately, early assessment of neurological status, which can lead to active withdrawal of life-sustaining treatment and thus mortality, is difficult [19, 23]. A good predictive method for the early days of post-arrest care would help to prevent treating unsalvageable patients for prolonged time and thus has extreme ethical importance. Current guidelines recommend determination of neuromarkers apart from clinical presentation, imaging diagnostics such as brain computed tomography or magnetic resonance imaging, as well as electrophysiological examination [18, 19, 24]. Most evidence for correlation of neuromarkers with anoxic brain damage after OHCA has been provided for NSE and S-100b with less evidence for S-100b [21]. Because of time dependent-changes in biomarker levels, a wide range of thresholds has been described in previous investigations with smaller population-sizes with cut-off values for NSE ranging from 8.8 to 80 μg/l and for S-100b ranging from 0.19 to 0.76 μg/l depending on classification of poor outcome, time of determination, and type of value such as cut-off, median or maximum level with good outcome [10]. Ranges for cut-off values for NSE from 5 to 82 μg/l and for S-100b from 0.18 to 0.30 μg/l have been reported during and after hypothermia, which were also calculated under the restrictions of small patient numbers [9]. Considering the large scattering range of biomarkers, poor neurological outcome is usually assumed in the majority of patients with NSE values >60 μg/l [24].

Several sub-analyses of the TTM-trial reported that elevated NSE [7] and S-100b [25] levels individually strongly predicted poor neurological outcome in that trial. More importantly, there were no major differences in median neuromarker levels in patients with good neurological outcome between normothermia and hypothermia. Furthermore, an analysis of both biomarkers in a hypothermia trial comparing duration of hypothermia of 24 to 48 hours [26] showed no alteration of prognostic reliability [27]. These findings suggest that there might be no difference in the level of those biomarkers at which they might have a prognostic meaning irrespective whether patients are treated with hypothermia or not. However, due to the large number of patients with withdrawal of life-support following prognostication in the TTM-trial [6], results might not be representative for patients without prognostication.

We determined cut-off values for NSE and S-100b in our hypothermia cohort without active prognostication and subsequent withdrawal of life-support, which were in the same range as described before without hypothermia [9]. They were associated with mortality and poor neurological outcome. Nevertheless, the false positive rate was still 10% for poor neurological outcome and 20% for mortality, which are both unacceptably high if the consequence of assessment will lead to withdrawal of life support. However, although the outliers showed similar tendencies in corresponding neuromarkers, we could not determine a sharp discrimination on the basis of these neuromarkers. We–like others- observed some patients with elevated biomarker values but good neurological outcome, and some with low values and poor outcome.

Ideally, a good predictive marker would identify patients at risk of developing a poor neurological outcome reliably with a low false predictive rate. However, of patients who achieved a good neurological outcome 20% had NSE above and 24% had S-100b above the ROC-determined cut-off for poor neurological outcome.

## Conclusions

We found similar cut-off values for NSE and S-100b under hypothermia to predict mortality and poor neurological outcome as described without hypothermia. In order to reach a still reliable prediction, both neuromarkers should never be used solitarily, but only in combination with other recommended methods such as EEG and imaging.

Therefore, higher cut-off values recommended by the guidelines should be used, as this lowers the risk of falsely predicting poor outcome. The low specificity of the cut-offs underlines the need for other predictive tools to either detect or exclude neurological damage early during post-reanimation care.

## Abbreviations

AMI: Acute Myocardial Infarction
CPC: Cerebral Performance Category
CPR: CardioPulmonary Resuscitation
HaCRA: Hannover Cardiac Resuscitation Algorithm
HACORE: HAnnover COoling Registry
ICU: Intensive Care Unit
NSE: Neuron-Specific Enolase
OHCA: Out-of-Hospital Cardiac Arrest
PAD: Peripheral Artery Disease
PCI: Percutaneous Coronary Intervention
ROSC: Return-Of-Spontaneous-Circulation
TTM: Targeted Temperature Management
